# Quantitative susceptibility mapping in pediatric neuroimaging: a systematic review of applications and advancements

**DOI:** 10.1007/s00247-026-06565-7

**Published:** 2026-03-09

**Authors:** Francesco Pacchiano, Mario Tortora, Serena Capasso, Mario Cirillo, Filippo Arrigoni, Fabio Tortora, Ferdinando Caranci, Kshitij Mankad, Lorenzo Ugga

**Affiliations:** 1 University of Naples Federico II, Via Sergio Pansini, 5, 80131 Naples, Italy; 2 University of Campania “Luigi Vanvitelli”, Caserta, Italy; 3 Ospedale dei Bambini Vittore Buzzi, Milan, Italy; 4 Great Ormond Street Hospital, London, United Kingdom

**Keywords:** Neonatal hemorrhage, Neurodevelopmental disorders, Pediatric neuroradiology, Quantitative susceptibility mapping

## Abstract

**Background:**

Quantitative susceptibility mapping (QSM) is an advanced magnetic resonance imaging (MRI) technique that quantifies tissue magnetic susceptibility, offering non-invasive insights into brain microstructure, including iron content and myelination. While extensively applied in adult neuroimaging, its use in pediatric populations is rapidly expanding.

**Purpose:**

This systematic review aims to provide a comprehensive overview of QSM applications in pediatric brain imaging, highlighting methodological advancements, diagnostic potential, and current limitations.

**Methods:**

A systematic literature search was performed using PubMed and Google Scholar up to April 2025. Inclusion criteria were original research articles written in English, involving only pediatric populations (0–17 years) and employing QSM in brain imaging. Twenty studies met eligibility criteria and were analyzed in terms of acquisition protocols, post-processing methods, study objectives, and main findings.

**Results:**

A systematic search on PubMed and Google Scholar found 54 QSM brain studies in children; after exclusions, 20 original research papers qualified for review and were quality-checked using Quality Assessment of Diagnostic Accuracy Studies version-2 (QUADAS-2). Most studies were recent (85% in the last 5 years), in Asia (55%, with China 35%), and used 3-tesla (T) MRI (80%). Typical imaging parameters: 8 echoes (TE=40 ms), slice thickness=2–2.5 mm, matrix often 256×256; Laplacian was the main phase-unwrapping method and variable-kernel sophisticated harmonic artifact reduction for phase data (VSHARP) the dominant background-field removal. Study aims clustered into improved detection, microstructural analysis, normative comparisons, clinical correlations, developmental patterns, and pathology tracking.

**Conclusion:**

QSM emerges as a valuable tool in pediatric neuroimaging, offering quantitative biomarkers for brain development, disease monitoring, and potential clinical translation. Despite promising results, challenges remain, including motion artifacts, lack of normative pediatric data, and methodological heterogeneity. Future research should focus on longitudinal designs, standardization of protocols, and integration with complementary imaging modalities. With further refinement, QSM has the potential to become an integral component of pediatric neuroradiological assessment.

**Graphical abstract:**

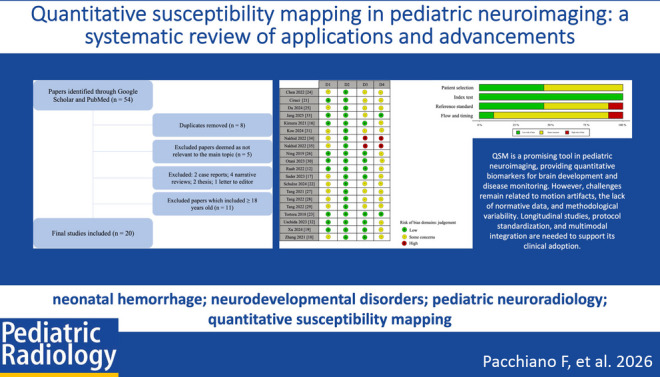

**Supplementary information:**

The online version contains supplementary material available at 10.1007/s00247-026-06565-7.

## Introduction

Quantitative susceptibility mapping (QSM) is an advanced magnetic resonance imaging (MRI) technique that quantifies the magnetic susceptibility of brain tissues, providing insights into iron and myelin distribution [1]. In recent years, QSM has gained increasing attention for its ability to non-invasively assess the distribution of paramagnetic substances such as iron and diamagnetic substances and structures such as calcium and myelin, thereby contributing to a deeper understanding of brain microstructure [2]. While extensively studied in adults, its application in pediatric neuroimaging is gaining interest due to the unique developmental processes occurring in the young brain [3]. QSM uses gradient-echo phase images to calculate the spatial distribution of magnetic susceptibility in tissue. Susceptibility is a substance-specific property, with paramagnetic materials producing positive χ and diamagnetic elements and structures having negative χ. The measured MRI phase is related to the underlying χ distribution by convolution with a dipole field kernel, solving an ill-posed inverse problem [4].

A typical pipeline includes:

- Image acquisition: 3-dimensional (D) multi-echo gradient-echo (GRE) data over the volume of interest to improve field estimation (echo times spaced <5 ms, with the last TE approximately 30–40 ms to match tissue T2*) [4].
- Brain masking/region extraction: Define a mask of the target region to exclude voxels with large susceptibility jumps (bone) that may compromise inversion [5].
- Phase unwrapping: Remove phase envelopes using region growing algorithms (path-based) or Laplacian unrolling [5].
- Background field removal: Methods include sophisticated harmonic artifact reduction for phase data (SHARP), regularization-enabled sophisticated harmonic artifact reduction for phase data (RESHARP), projection onto dipole fields, or Laplacian boundary value to obtain the local field [6].
- Dipole inversion: Several methods can be used for dipole inversion, such as truncated k-space division, enabled morphology, iterative least squares, and iterative Tikhonov regularization. Streaking artifacts can be a problem with these methods [7–9].

Artificial intelligence (AI) plays a role in the acceleration of data acquisition [10] or in simplifying the QSM processing [11]. In the first 2 years of life, the brain undergoes rapid maturation, including myelination, iron deposition, and structural reorganization. QSM offers a non-invasive means to monitor these processes, revealing age-specific variations in tissue composition [12]. For instance, studies have shown that the magnetic susceptibility of cerebrospinal fluid decreases progressively from neonates to adults, reflecting changes in macromolecular content [13]. Additionally, QSM has identified distinct susceptibility contrasts in white matter regions, correlating with myelination patterns and iron content [14]. Clinically, QSM has been utilized to assess deep gray matter development [15], detect abnormal iron accumulation in conditions like neurodegeneration with brain iron accumulation [16], and evaluate brain injury recovery, such as in pediatric trauma [17]. In pediatric settings, QSM can provide valuable insights into various neurological conditions, including cerebral palsy [18] and epilepsy [19]. However, interpreting susceptibility data in children is complex due to the rapid and dynamic changes occurring in brain development and age-related physiological variability. Moreover, the lack of standardized protocols and age-specific reference data limits the ability to make systematic comparisons across studies [20]. This review aims to provide a critical overview of the use of QSM in the pediatric brain, discussing the physical principles of the technique, its diagnostic potential, methodological challenges, and future perspectives. The goal is to outline the emerging role of QSM as a quantitative tool in pediatric neuroradiology and to encourage the development of dedicated studies in this vulnerable yet crucial population for understanding the human brain.

## Materials and methods

**Fig. 1:**
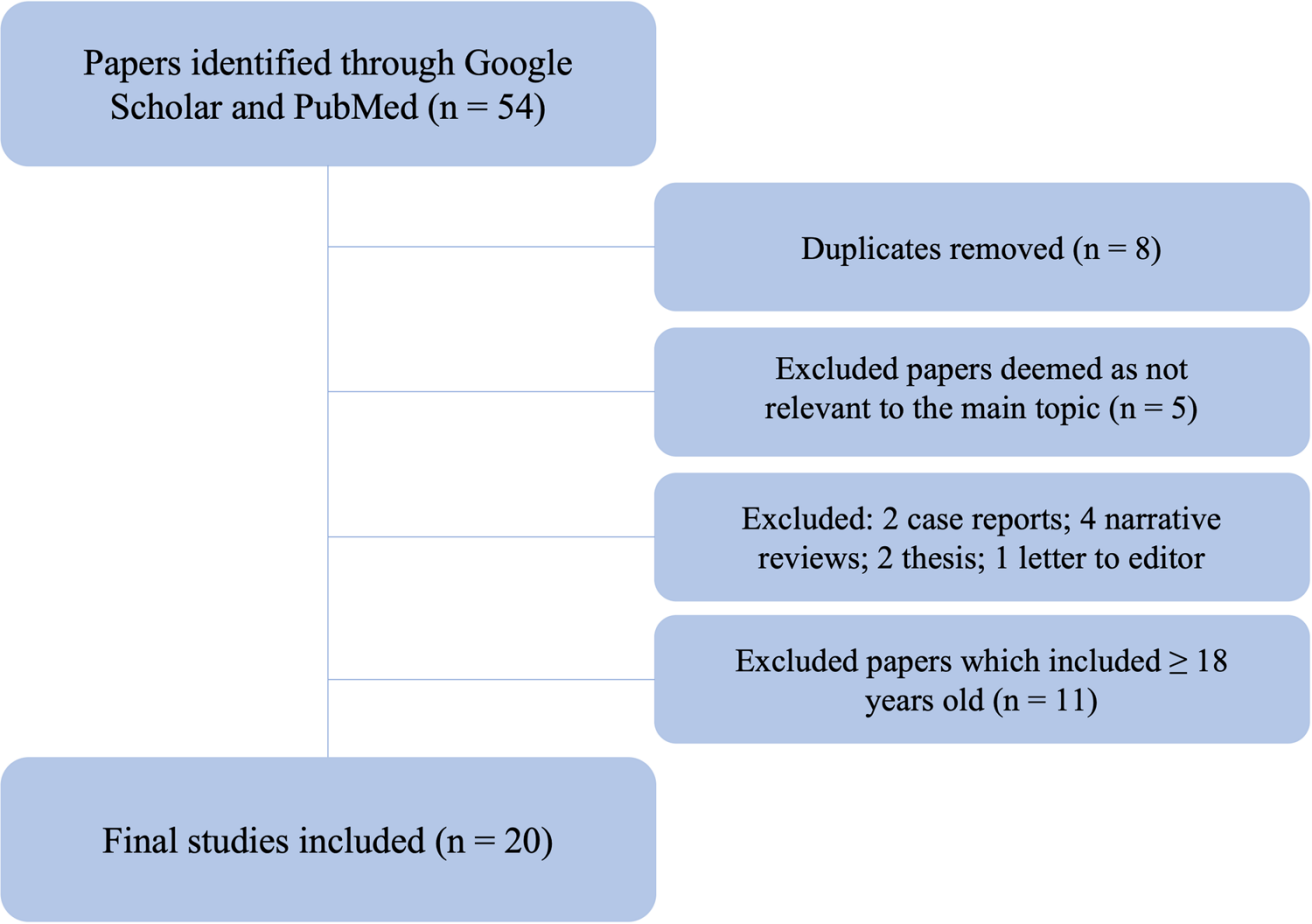
Preferred Reporting Items for Systematic Reviews and Meta-Analyses (PRISMA) flow diagram summarizing the systematic review study selection process

A systematic literature search was conducted to identify relevant studies reporting on the use of quantitative susceptibility mapping (QSM) in the pediatric brain. Two widely recognized scientific databases, PubMed and Google Scholar, were queried using the search terms: “QSM” OR “Quantitative susceptibility mapping” AND “Children” OR “Pediatric” AND “Brain”. No restriction was applied to the year of publication to ensure an exhaustive retrieval of relevant literature up to April 2025. Two researchers, F.C. (with more than 25 years’ experience in neuroradiology) and L.U. (with 10 years’ experience in neuroradiology and pediatric neuroradiology) determined the eligibility of the articles through title and abstract evaluation. Studies were eligible for inclusion if they met the following criteria: original research articles; written in English; only involving in their cohort patients with a pediatric population (0–18 years); employed QSM as part of the neuroimaging protocol focused on brain imaging. The following exclusion criteria were applied: case reports; review articles; editorials, letters to the editor, or conference abstracts without full data; doctoral or specialist theses; studies that did not use QSM or did not include a pediatric cohort. Figure 1 shows the “Preferred Reporting Items for Systematic Reviews and Meta-Analyses” (PRISMA) flow chart used in this review.

## Results

**Fig. 2:**
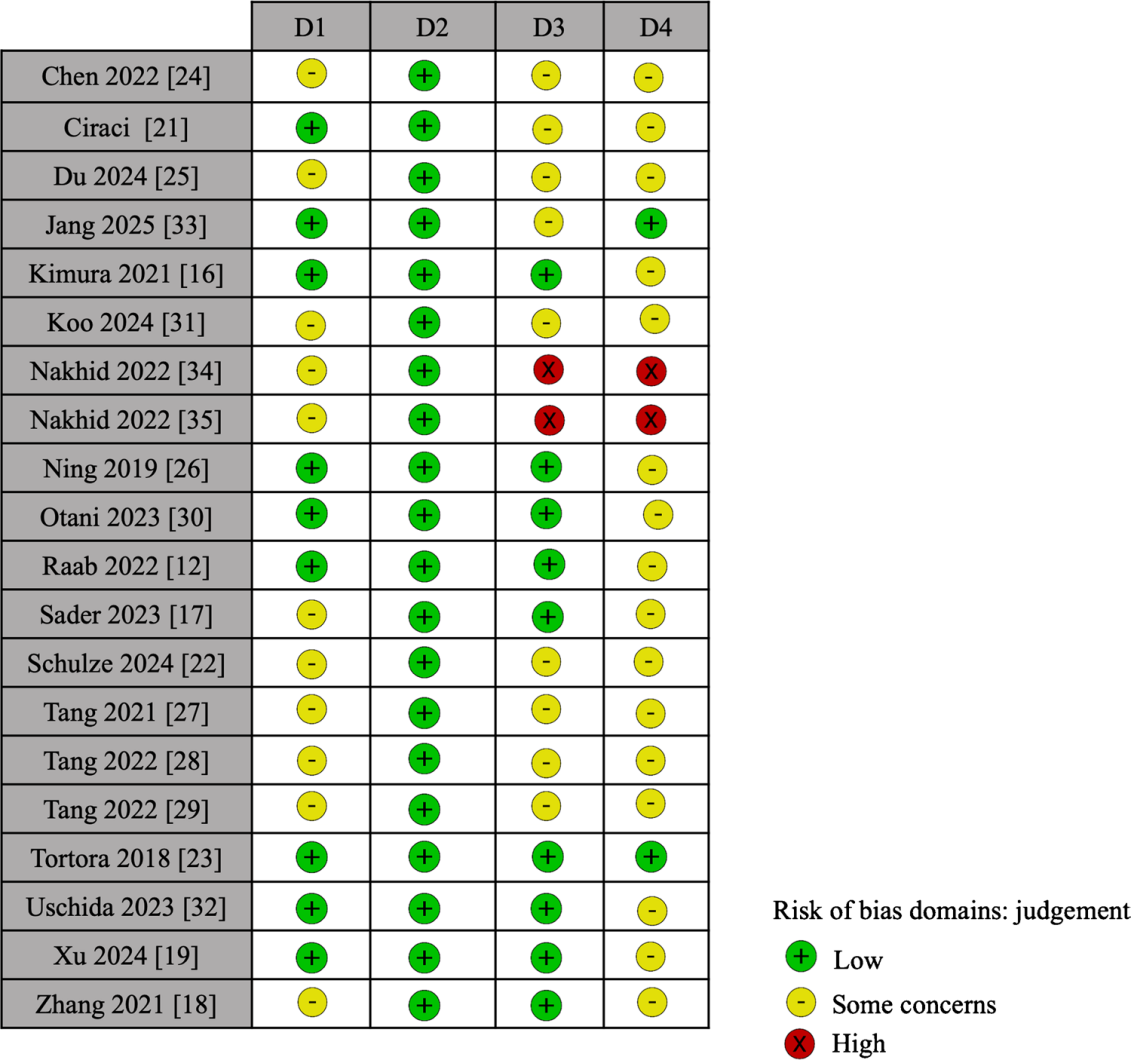
Quality Assessment of Diagnostic Accuracy Studies (QUADAS)−2. Each domain is rated for risk of bias (low, high, or some concerns). *D1*, patient selection; *D2*, index test; *D3*, reference standard; *D4*, flow and timing

**Fig. 3:**
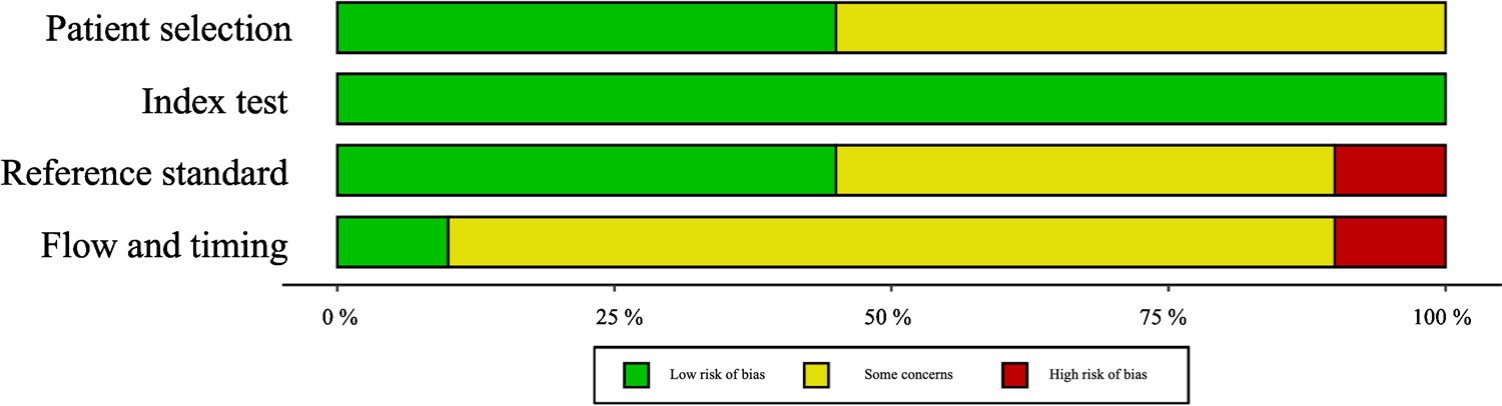
Quality Assessment of Diagnostic Accuracy Studies (QUADAS)−2. The tool evaluates risk of bias across four domains: patient selection, index test, reference standard, and flow and timing

A systematic literature search was conducted using the keywords “Quantitative susceptibility mapping (QSM),” “children,” and “brain” across two leading biomedical databases—PubMed and Google Scholar—resulting in an initial yield of 54 articles. After the removal of 8 duplicates and 5 articles irrelevant to the topic, the remaining studies were screened based on predefined inclusion criteria (original research involving completely or partially a pediatric population) and exclusion criteria (case reports *n*=2, review articles *n*=4, doctoral/specialist theses *n*=3, and letters to the editor *n*=1). This process yielded 31 original research articles. We systematically checked if each paper included only a population with age <18 years old and excluded the remaining; this inclusion criteria led to the exclusion of 11 papers, and the final inclusion of the remaining 20 defined eligible for systematic review (Table 1). Quality assessment was then performed by L.U. through the QUADAS-2 system (Figs. 2 and 3 and Supplementary Material).

**Table 1:** Characteristics of the studies included in this systematic review of quantitative susceptibility mapping in the pediatric brain, divided per regions: Europe, China, Japan/South-Korea, and USA/Canada. *3D*, 3-dimensional; *ADHD*, attention-deficit/hyperactivity disorder; *ASD*, autism spectrum disorder; *AUC*, area under the curve; *BBB*, blood-brain barrier; *CBF*, cerebral blood flow; *DKI*, diffusion kurtosis imaging; *DTI*, diffusion tensor imaging; *FA*, fractional anisotropy; *FOV*, field of view; *GMH-IVH*, germinal matrix hemorrhage – intraventricular hemorrhage; *GP*, globus pallidus; *GRE*, gradient echo; *LBV*, Laplacian boundary value; *MEDI*, morphology-enabled dipole inversion; *MF*, magnetic field strength (tesla); *MRI*, magnetic resonance imaging; *MSV*, magnetic susceptibility value; *n*, number; *ND*, not defined; *PDF*, projection onto dipole fields; *PMA*, post-menstrual age; *QSM, *quantitative susceptibility mapping; R2*, apparent transverse relaxation rate; *RESHARP*, regularization-enabled SHARP; *ROC*, receiver operating characteristic; *SWI*, susceptibility-weighted imaging; *SHARP*, sophisticated harmonic artifact reduction for phase data; *ST*, slice thickness (mm); *TE*, echo time (ms); *UTE*, ultra-short echo time; *VSHARP*, variable-kernel SHARP; *WM*, white matter. “**Aim category**” indicates the primary study focus: *typical brain development and aging* (normative iron and myelin changes), *neurodevelopmental disorders* (autism spectrum disorder, attention-deficit/hyperactivity disorder), *epilepsy-related pathology* (including self-limited epilepsy with centrotemporal spikes), *neurodegenerative disorders* (such as neurodegeneration with brain iron accumulation), *vascular or hemorrhagic disorders* (intracranial hemorrhage, calcification), *metabolic or psychiatric disorders* (including prenatal alcohol exposure), *post-treatment microstructural change* (including therapy in cerebral palsy), and *trauma/concussion studies*. Image-processing methods are written in full: Laplacian phase-unwrapping, projection onto dipole fields for background field removal, Laplacian boundary value, morphology-enabled dipole inversion, iterative least squares, and truncated k-space division

Author	Year	Nationality	No. of patients	MR sequences	QSM parameters	Post-processing	Aim	Outcome		Aim category
Europe										
Ciraci et al. [21]	2017	Turkey	16	QSM vs SWI	MF: 1.5 T; ST: 2.5 mm; echoes: 5–8.3.3 ms to 40.7 ms, echo-spacing: 6.48 ms; resolution: 1.146×1.338×2.5 mm^3; matrix: 192×154; FOV: 220×206; flip angle: 25	Phase unwrapping: ND; background field removal: projection onto dipole fields	Differ brain calcifications and hemorrhage	QSM had a high sensitivity (>84.7%) and specificity (>98.3%) in differentiating		Vascular/hemorrhage
Raab et al. [12]	2022	Germany	74	QSM	MF: 3 T; ST: 2 mm; echoes: 8 - 3.6 ms to 45 ms- echo-spacing: 5.91 ms; resolution: 1×1×2 mm^3; matrix: 256×256; FOV: 240×240; flip angle: 15	Phase unwrapping: ND; background field removal: ND	Investigate age-related iron deposition in the deep gray matter in children using quantitative susceptibility (QSM) and R2* mapping	Susceptibility changes in the globus pallidus, caudate nucleus, and putamen strongly correlated with age, with R2* increasing faster than QSM in the first 2 years, suggesting early combined effects of iron deposition and myelination, later dominated by iron accumulation		Typical brain development and aging
Schulze et al. [22]	2024	Germany	111	QSM	MF: 3 T; ST: ND; echoes: 6- 7.38 ms to 44.27 ms-echo-spacing: 7.38 ms; resolution: ND; matrix: 204×224×160; FOV: ND; flip angle: 12	Phase unwrapping: Laplacian; background field removal: VSHARP	Brain iron in subcortical structures in children with attention-deficit/hyperactivity disorder	No differences with normal cohort		Attention-deficit/hyperactivity disorder
Tortora et al. [23]	2018	Italy	127	QSM	MF: 1.5 T; ST: ND; echoes: ND; resolution=0.234×0.234, matrix size: 512×512, FOV: 120×120, flip angle: 15	Phase unwrapping: ND; background field removal: ND	Evaluate magnetic susceptibility of normal-appearing white (WM) and gray matter regions in preterm neonates with and without GMH-IVH	Paramagnetic shifts in normal-appearing WM of neonates with severe GMH-IVH likely reflect iron buildup from hemoglobin diffusion into periventricular tissue		Vascular/hemorrhage
Asia										
Chen et al. [24]	2022	China	102	QSM	MF: 3 T; ST: 2 mm; echoes: 8 - first eco 4.4 -echo-spacing ND; resolution=1×1×2 mm^3; matrix size: 256×256; FOV: 25.6×25.6; flip angle: 20	Phase unwrapping: ND, background field removal: ND	Differences in attention-deficit/hyperactivity disorder and normal cohort	Iron deficiency was found in key brain regions in children with attention-deficit/hyperactivity disorder, with the left anterior cingulum showing a positive link to symptom severity (*r*=0.326, *P*<0.05)	Attention-deficit/hyperactivity disorder	
Du et al. [25]	2024	China	33	QSM	MF: 3 T; ST: 1 mm; echoes: 8 - 3.2 ms to 19.8 ms – echo-spacing: 2.3 ms; resolution=1×1×1 mm^3, matrix size: 256×256, FOV: 256×256, flip angle: 12	Phase unwrapping: Laplacian; background field removal: VSHARP	Susceptibility values in specific brain regions correlated with disease severity and neurodevelopmental status in children with autism	ROC analysis revealed higher AUCs for frontal WM susceptibility in ASD, while right globus pallidus susceptibility positively correlated with fine motor scores	Autism spectrum disorder	
Ning et al. [26]	2018	China	87	QSM	MF: 3 T; ST: 2 mm, echoes: 6- 7 ms to 46 ms, echo-spacing: 6.5 ms; resolution: 0.703×0.703×2 mm^3; matrix: 256×256; FOV: 180×180; flip angle: 20	Phase unwrapping: Laplacian; background field removal: SHARP	Quantify variation of brain iron-related susceptibility during development	Positive correlations of susceptibility with both referenced iron concentration and age were found	Typical brain development and aging	
Tang et al. [27]	2021	China	80	QSM	MF: 3 T; ST: 3 mm; echoes: ND; resolution=ND; matrix size: ND; FOV: 24; flip angle: 20	Phase unwrapping: ND, background field removal: ND	Susceptibility values in children with autism vs controls	Children in the study group showed significantly lower magnetic susceptibility in key brain regions compared to controls—starting in the caudate, dentate, and splenium at ages 2–3, and extending to multiple additional areas from ages 3 to 6 (*P*<0.05)	Autism spectrum disorder	
Tang et al. [28]	2022	China	102	QSM	MF: 3 T; ST: 3 mm; echoes: ND; resolution; ND; matrix: ND; FOV: 24; flip angle: 20	Phase unwrapping: ND, background field removal: ND	Differences in iron levels between attention-deficit/hyperactivity disorder children and normal cohort	The volume of frontal lobe and hippocampus of children with attention-deficit/hyperactivity disorder was lower than that of healthy children (*P*<0.05). Iron content in brain areas such as globus pallidus, caudate nucleus, hippocampus, and putamen could distinguish children with attention-deficit/hyperactivity disorder (area under the curve [AUC]>0.5, *P*<0.05)	Attention-deficit/hyperactivity disorder	
Tang et al. [29]	2022	China	120	QSM, DKI, 3D-PCASL	MF: 3 T; ST: 3 mm; echoes: ND; resolution: ND; matrix: ND; FOV: 24; flip angle: 20	Phase unwrapping: ND, background field removal: ND	Differences between autistic children and normal cohort	The values of CBF, QSM, and DKI in frontal lobe, temporal lobe, and hippocampus could distinguish autistic children (AUC>0.5, *P*<0.05)	Autism spectrum disorder	
Xu et al. [19]	2024	China	50	QSM and DTI	MF: 3 T; ST: 2 mm; echoes: 10 - 3.34 ms to 25.16 ms - echo-spacing: 2.43 ms; resolution=0.85×0.85; matrix size: 256×256; FOV: 220; flip angle: 12	Phase unwrapping: Laplacian; background field removal: VSHARP	Altered white matter regions in self-limited epilepsy with centrotemporal spikes patients can be detected by combining DTI and QSM, revealing microstructural and susceptibility changes linked to epileptogenic areas	DTI and QSM revealed widespread white matter changes in self-limited epilepsy with centrotemporal spikes patients, with overlapping alterations in the corona radiata where FA values were inversely correlated with magnetic susceptibility	Epilepsy related	
Kimura et al. [16]	2020	Japan	14	QSM	MF: ND; ST: ND; echoes: 7TE- 6.5 to 44.9 ms-echo-spacing: 6.4 ms; resolution: 1.0×1.0×2.0 mm^3; matrix: 240×256; FOV: 240×256; flip angle: 20	Phase unwrapping: ND; background field removal: ND	Iron levels useful to differentiate between beta-propeller protein–associated neurodegeneration and control groups	The QSM values of the Globus pallidus and substantia nigra were significantly higher in the patients compared to the controls	Neurodegenerative	
Otani et al. [30]	2023	Japan	138	QSM	MF: 3 T; ST: 2 mm; echoes: 4 - 10 ms to 40 ms – echo-spacing: 10 ms; resolution: 1×1×2 mm^3; matrix-size: 240×240; FOV: 240×240; flip angle: 15	Phase unwrapping: Laplacian, background field removal: ND	Differences in iron accumulation during development in patients with normal development and delayed	Magnetic susceptibility values in deep gray matter increased with age ≤ 1,000 days. The normal development group showed higher susceptibility values than the delayed development group at early postnatal age (PMA ≤ 285 days)	Typical brain development and aging	
Koo et al. [31]	2023	South Korea	23	QSM	MF: 3 T; ST: ND; echoes:4 - TE: 4.92 ms to 19.68 ms-echo-spacing: 4.92 ms; resolution: 0.78×0.78×2 mm^3; matrix: 256×256×80; FOV: 199.68×199.68×160; flip angle: ND	Phase unwrapping: Laplacian; background field removal: VSHARP	Iron levels in autism spectrum	The verbal comprehension index significantly correlated with the left-caudate MSV (*r*=0.420, *P*=0.046) and the perceptual organization index significantly correlated with the right-globus-pallidus MSV (*r*=0.414, *P*=0.049)	Autism spectrum disorder	
Uchida et al. [32]	2023	Japan	78	QSM and DP-PCASL	MF: 3 T; ST: ND; echoes: 12- 3.8 ms to 45.6 ms-echo-spacing: 3.8 ms; resolution: 1×1×1 mm^3; matrix: 192×192; FOV: 192×192; flip angle: 15	Phase unwrapping: Laplacian; background field removal: VSHARP	Evaluate relationship between iron dynamics and BBB function in development	Rapid susceptibility increases were seen in deep gray matter—most notably in the globus pallidus—during the first 2 years. Susceptibility changes followed a sigmoidal pattern relative to BBB water exchange dynamics, reflecting region-specific iron accumulation	Typical brain development and aging	
America										
Jang et al. [33]	2024	USA	65	QSM	MF: 3 T; ST: ND; echoes: 7 TE- 5 to 44 ms-echo-spacing: 5.5 ms; resolution=0.38×0.4×2 mm3 or 0.5×0.63×2 mm3, matrix size: 421×400×50, FOV: 160×160×100 mm^3, flip angle: 20	Phase unwrapping: ND; background field removal: VSHARP	Quantified the separate contributions of positive and negative susceptibilities within each voxel to more accurately assess myelin and iron levels during early brain development in newborns	White matter showed lower positive and higher negative susceptibility than cortical and deep gray matter. Age-linked susceptibility changes appeared in the putamen and GP, with occipital lobes distinctively diverging from frontal in both measures	Typical brain development and aging	
Nakhid et al. [34]	2022	Canada	59	QSM	MF: 3 T; ST: ND; echoes: 10- 4.5 ms to 52.2 ms-echo-spacing: 4.77 ms; resolution: 0.94×1.17×1.90 mm^3; matrix: 255×205×96; FOV: 240×240×182.4; flip angle: 10	Phase unwrapping: best path; background field removal: RESHARP	Brain volumes and susceptibility could be associated with internalizing or externalizing symptoms in youth with prenatal alcohol exposure	Susceptibility in the nucleus accumbens was negatively associated with internalizing problems, while amygdala susceptibility was positively associated with internalizing problems across groups	Psychiatric	
Nakhid et al. [35]	2022	Canada	64	QSM	MF: 3 T; ST: ND; echoes: 10 - 4.5 ms to 52.2 ms – echo-spacing: 5.3 ms; resolution=0.94×1.17×1.90 mm3; matrix size: 255×205×96; FOV: 240×240×182.4, flip angle: 10	Phase unwrapping: best path; background field removal: RESHARP	Prenatal alcohol exposure, brain iron	No significant group differences in susceptibility emerged after correction, though thalamic susceptibility was higher in the prenatal alcohol exposure group before correction (*P*=0.032, *q*=0.230). Thalamic susceptibility showed no link to IQ	Metabolic	
Sader et al. [17]	2022	Canada	371	QSM	ND	ND	QSM in children following concussion or orthopedic injury (OI), to assess QSM performance as a diagnostic and prognostic biomarker	Higher QSM values in the frontal white matter were linked to worse post-concussion symptoms	Trauma	
Zhang et al. [18]	2021	USA	8	QSM and DTI	MF: 3 T; ST: ND; echoes: 16-3 ms to 36.9 ms -echo-spacing: 2.12 ms; resolution=1×1×1 mm^3; matrix size: 192×192×120, FOV: 192×192×120; flip angle: 20	Phase unwrapping: Laplacian; background field removal: V-SHARP	Mechanism of the observed motor function improvement and brain connectivity increase in cerebral palsy patients who received autologous UCB stem-cell therapy	High responders showed markedly greater diamagnetic Δχ and ΔMSA in the periventricular CST (*P*=0.003, *P*=0.006), alongside increased FA	Cerebral palsy, post-treatment changes in microstructure

Each study was evaluated systematically according to key variables, including author, year of publication, country of origin, sample size, QSM acquisition protocols, post-processing methods, study objectives, and main findings. To facilitate comparative and statistical analysis, study objectives were classified into standardized thematic categories: improved detection, microstructural analysis, comparisons with normative cohorts, clinical correlations, developmental trajectories, and pathological development.

Among the 20 included studies, 85% (17/20) were published within the last 5 years. A regional analysis revealed that 55% (11/20) of studies originated from Asia, with China being the most represented country (35%, 7/20). The median sample size across studies was 86.1 (minimum=8, maximum=371), with 7 studies (35%) including a cohort of 100 participants or more.

In terms of magnetic resonance imaging (MRI) field strength, most studies (80%, 16/20) utilized 3-T scanners, while 2 studies (10%) employed 1.5-T systems.

Out of the 20 articles, 15 provided valid data regarding the number of echoes used in QSM sequences. The mean number of echoes was 8.1. The minimum number of echoes used was 4, while the maximum reached 16. Echo spacing data was available in 14 articles. The average echo-spacing was approximately 5.27 ms. The minimum spacing was 2.12 ms, while the maximum was 10.0 ms. Final TE data could be derived from 14 articles where both echo count and spacing were available. The mean final TE was 39.74 ms. The shortest final TE was 19.68 ms, and the longest reached 52.20 ms. Approximately 71% of the studies with valid data had a final TE less than or equal to 40 ms.

Slice thickness was available in only 50% (10/20) of the total articles; the mean slice thickness was 2.25 mm with a minimum of 1.0 mm and a maximum of 3.0 mm. Sixty percent of the studies with valid slice thickness data used a thickness between 2.0 mm and 2.5 mm. Resolution was possible to be extracted in 75% (15/20) of the papers; only 5 articles reported iso-voxel resolutions. Matrix parameter was directly available in 19 papers and was possible to calculate in another 6; matrix was then possible to be evaluated in 70% of papers (14/20); the most frequently used was 256×256 in around 30% of the total papers. FOV was possible to be extracted in 75% (15/20) of the papers. Flip angle was available in 90% (18/20) of papers; the minimum was “10”; the maximum was “25”; the mean was “17”; and the most frequently used was “20” in 40% (8/20).

Regarding post-processing, the phase-unwrapping methods were available or possible to be determined by the reviewing authors in 50% (10/20) of the papers. Among the identified valid methods, Laplacian was the most prevalent, occurring 8 times, which corresponds to 40% (8/20) of the total dataset and 80% (8/10) of all available data, indicating a dominance of the Laplacian approach among the methods explicitly provided. The Best-path method appeared only twice with a percentage of 10% (2/20). Regarding background field removal, it was possible to be evaluated in 55% of papers (11/20); the most frequently reported method was VSHARP, which appeared in 35% (7/20) of the total dataset and in around 64% (7/11) of the papers where the data was available, highlighting it as the dominant method in the sample.

The focus of the articles was systematically categorized into thematic domains to facilitate comparison and identify prevailing trends in QSM applications in pediatric neuroimaging. Table 1 summarizes the data and characteristics of the reviewed articles.

A significant proportion of the studies: 25% (5/20) centered on typical brain development and aging, specifically exploring changes in iron and myelin content across infancy, childhood, and adulthood. Approximately 20% (4/20) of the studies investigated autism spectrum disorder examining alterations in brain iron levels among children with autism spectrum disorder and their potential associations with cognitive and motor function. Around 15% (3/20) focused on attention-deficit/hyperactivity disorder, assessing differences in brain iron between affected individuals and controls, as well as correlations with symptom severity. A further 5% (1/20) of the studies addressed epilepsy, with one concentrating on focal cortical dysplasia and another on self-limited epilepsy with centrotemporal spikes. Another 5% (1/20) examined alterations in brain iron in the context of neurodegenerative disorders. Nearly 10% (2/20) investigated vascular brain disorders, including differentiating calcium vs blood products and intracranial hemorrhages. Metabolic disorders, such as prenatal alcohol exposure accounted for 5% (1/20), while psychiatric conditions, particularly internalizing and externalizing symptoms associated with prenatal alcohol exposure, were also the focus of 5% (1/20). Post-treatment changes in brain microstructure in patients with cerebral palsy and their relationship to clinical outcomes were explored in 5% (1/20) of the studies. Another 5% (1/20) investigated brain alterations following trauma, specifically post-concussion effects.

## Discussion

This systematic review underscores the rapidly expanding application of quantitative susceptibility mapping (QSM) in pediatric neuroimaging, with 85% of included studies published within the last 5 years, reflecting the escalating interest in susceptibility-based biomarkers of brain development. The physiologic basis of QSM—its sensitivity to the magnetic properties of iron, myelin, calcium, and other microstructural contributors—provides a unique contrast mechanism that complements and extends conventional magnetic resonance imaging (MRI). In the developing brain, where dynamic iron redistribution, oligodendrocyte maturation, and axonal myelination evolve in tightly regulated trajectories, QSM offers a quantitative window into processes that were previously only inferable through indirect imaging proxies.

Normative pediatric studies consistently delineate age-dependent susceptibility increases across deep gray matter nuclei, particularly within the globus pallidus, putamen, and caudate. These trajectories reflect the combined influence of iron accumulation and myelin-related susceptibility changes and appear to follow nonlinear maturational curves across infancy, childhood, and adolescence. Importantly, QSM-derived normative datasets provide a quantitative scaffold against which pathological deviations can be assessed. However, the scarcity of large longitudinal cohorts remains a significant limitation; current datasets are often cross-sectional and regionally restricted. Future initiatives should prioritize multiethnic longitudinal sampling to account for demographic and environmental modifiers of susceptibility values, ensuring generalizability and clinical interpretability [12, 26, 30, 33].

Neurodevelopmental disorders constitute the most mature area of pediatric QSM research to date. In autism spectrum disorder (ASD), several studies have demonstrated regionally reduced magnetic susceptibility in subcortical structures and white matter pathways, with some findings correlating with cognitive, motor, or behavioral outcomes [25, 27, 29, 31]. These patterns may reflect altered iron metabolism, atypical myelin development, or disrupted neuroinflammatory pathways. Nevertheless, the heterogeneity of findings underscores the need for harmonized pipelines, larger samples, and the integration of QSM with genetic, metabolic, and behavioral phenotyping. In attention-deficit/hyperactivity disorder (ADHD), preliminary evidence suggests altered iron-related susceptibility within the basal ganglia [24, 28], although results remain inconsistent due to small sample sizes, divergent reconstruction methods, and substantial clinical heterogeneity [22]. Standardizing susceptibility quantification and improving motion control will be essential to resolving these inconsistencies.

Clinical applications beyond neurodevelopmental syndromes illustrate QSM’s broader diagnostic and prognostic potential. In pediatric epilepsy, QSM enhances lesion conspicuity by revealing subtle iron-related abnormalities and microstructural changes not identifiable on T1- or T2-weighted imaging. These susceptibility abnormalities may reflect hemosiderin deposition, altered myelin architecture, or chronic inflammatory changes, thereby offering valuable information for pre-surgical mapping and lesion characterization [19]. In cerebral palsy, susceptibility shifts following autologous cord-blood therapy have demonstrated diamagnetic changes consistent with increased myelin content, highlighting QSM’s potential as a quantitative biomarker of neuroregenerative treatment response [35]. Early investigations in pediatric traumatic brain injury and concussion reveal that susceptibility alterations within frontal white matter pathways may serve as predictors of persistent post-concussive symptoms, although these early findings require prospective, multi-center validation [17].

Several additional domains remain underrepresented but show promising early signals. These include pediatric neurodegeneration [16], cerebrovascular and perfusion-related disorders such as arteriovenous malformations or moyamoya disease [21, 23], and metabolic or psychiatric conditions including prenatal alcohol exposure [34, 35]. Given QSM’s sensitivity to iron dysregulation and microstructural integrity, its application across these domains may uncover novel biomarkers of disease severity, chronicity, or treatment responsiveness.

Despite encouraging progress, several barriers impede the full clinical translation of QSM in pediatric imaging. Substantial methodological heterogeneity—including differences in echo times, multi-echo acquisition design, field strengths, unwrapping and background-field removal algorithms (e.g., Laplacian-based methods, SHARP/RESHARP), and dipole inversion techniques—limits cross-study comparability. Pediatric susceptibility measurements are especially sensitive to motion artifacts; thus, motion-robust acquisition strategies (e.g., radial sampling, navigator echoes, real-time prospective motion correction) and AI-assisted reconstruction will be essential to ensure reproducible quantification. The dominance of studies conducted on 3-T MRI systems reflects the need for high signal-to-noise ratios when probing subtle susceptibility variations in small pediatric structures. Moreover, the geographic concentration of studies—largely from Asia, particularly China—highlights the need for broader international collaboration to ensure demographic, environmental, and ethnocultural diversity in normative references.

Looking ahead, critical priorities must be addressed to enable the routine clinical use of QSM in pediatric neuroradiology. First, acquisition and reconstruction workflows require standardization across vendors and institutions, supported by consensus guidelines and phantom-based quality assurance programs. Second, multi-center longitudinal studies that follow children from infancy through adolescence are essential for mapping normative susceptibility trajectories and identifying the developmental windows most sensitive to pathological divergence. Third, integrating QSM with multimodal approaches—including diffusion MRI, myelin-sensitive imaging, resting-state fMRI, MR spectroscopy, and emerging metabolic imaging tools—may offer a more comprehensive understanding of neurodevelopmental dynamics. Finally, building open-access, age-stratified normative databases and implementing automated, region-specific quantification pipelines will accelerate QSM’s adoption as a practical biomarker for diagnosis, prognosis, and monitoring therapeutic response.

So, QSM represents a powerful and increasingly mature tool in pediatric neuroimaging, capable of quantifying biologically meaningful aspects of brain development and pathology that remain inaccessible to conventional MRI. Realizing its clinical potential will require coordinated methodological standardization, expanded longitudinal datasets, multimodal integration, and widespread international collaboration. With these steps, QSM is poised to evolve from a research-focused technique to a clinically actionable biomarker across a broad spectrum of pediatric neurological disorders.

## Conclusion

Quantitative susceptibility mapping (QSM) is redefining pediatric neuroimaging by offering a sensitive, non-invasive window into brain development and pathology. As this review illustrates, QSM in pediatric imaging was mainly used to evaluate normal changes in the infant/child brain, the main clinical applications were related to neurodevelopmental disorders, leaving a wide space for the study of neuroinflammatory and neurodegenerative disorders. With further standardization and longitudinal research, QSM is set to become an indispensable tool in the evolving landscape of pediatric brain imaging.

## Supplementary information

Below is the link to the electronic supplementary material.

ESM 1 (XLSX 9.21 KB)

## Data Availability

No datasets were generated or analysed during the current study.
